# Editorial

**Published:** 1912-11

**Authors:** 


### EDITORIAL

The Eusiness Office of the *Journal-Record* is 23 West Alabama Street.

The Editorial Office is 1014-15 Atlanta National Bank Building.

Address all Business Communications to *Journal-Record of Medicine*, 23 West Alabama Street.

Make remittances payable to *The Journal-Record of Medicine.*

On matters pertaining to the Editorial and Original Communications, address Edgar G. Ballenger, M. D., Atlanta, Ga.

Reprints of Original Articles will be furnished at cost price. Requests for the same should always be made in THE MANUSCRIPT.

We will present, postpaid, on request to each contributor of an original article, twenty (20) marked copies of *The Journal-Record of Medicine* containing such article.

### SOUTHERN MEDICAL ASSOCIATION

The recent meeting of the Southern Medical Association, held in Jacksonville November 12 13 and 14, was the most successful in the career of this asscociation. Representative men from all of the Southern States were present and were royally entertained by the Jacksonville physicians and citizens. In fact it is most unusual for an association to receive such an elaborate banquet as that tendered the attending physicians on the second night.

The class of scientific work was the bets ever presented to this society and the physicians who were not present suffered a distinct loss.

Lexington, Kentucky, was selected as the place of meeting for the next annual meeting.

The following officers were elected for the ensuing year: President, Dr. Frank A. Jones, Memphis, Tenn.; First Vice-President, Dr. Stuart McGuire, Richmond, Va.; Second Vice-President, D. J. D. Love, Jacksonville, Fla.; Secretary-Treasurer, Dr. Seale Harris, Mobile, Ala.

Among the resolutions presented the following is most commendable:

*To The Honorable, The Congress of the United States*:

Whereas, The Southern Medical Association, a non-political, non-partisan organization of Southern physicians, whose sole object is the advancement of medical education in the South and the distribution of medical knowledge among the people, desires in every possible way to move towards the absolute eradication of malaria, which, every year costs our country many millions of dollars and precious lives; and, whereas, the accomplishment of this benefaction to the nation and to humanity will demand powers and resources far beyond those available to any organization of our citizens; therefore,

Resolved, That the Congress of the United States of America be and is hereby respectfully urged to create a commission for the investigation of malaria, with a view to recommending to the President and Congress such measures as will in their judgment be most practically efficient in extirpating the disease.

Resolved, That such commission should be amply provided with funds and invested with liberal powers, so that its action may be comprehensive and effectual and it may not be hampered in any direction, whether as to men, means or territories.

Resolved, That details of medical officers of the army, the navy, and the public health and marine hospital service should be placed by the president at the service of this commission.

Resolved, That this association most respectfully urges that the matter should be attended to without delay, as every hour of every day somewhere, adds to the numbers destroyed by this eradicable disease.

Resolved, That the secretary-treasurer of the association shall send a copy of these resolutions to the President, to the President-elect and to every member of the houses of Congress.

Resolved, That this association declares its desire and willingness to co-operate through its membership in facilitating the object contemplated.

### ORGANIZATION, POWERS AND DUTIES OF HEALTH AUTHORITIES

The Treasury Department has issued a valuable analysis of the laws and regulations relating to the health authorities of the United States. This work has been done by Assistant Surgeon General J. W. Kerr and should afford a splendid summary of the subject for all who are interested in health laws and authorities. Kerr says three requisits are indespensable to properly protect the public health:

1. Sanitary legislation in harmony with the latest progress in hygiene. 2. Efficient organization for the enforcement of law. 3. Adequate appropriations to bear the expense involved.

The first attempts at public health legislation were made in Virginia in 1639, the object being to regulate the practice of medicine. The first city health officer was appointed in Baltimore in 1793 on account of fear of yellow fever which afflicted Philadelphia that same year.

One of the most important duties of state boards which has been receiving much special attention during recent years is the education of the masses in the preservation of health and the prophylaxis of disease.

If those who are interested in framing and passing medical laws would study carefully this digest we would not have so many ill-advised or ignored laws.

